# Structural basis of fast- and slow-severing actin–cofilactin boundaries

**DOI:** 10.1016/j.jbc.2021.100337

**Published:** 2021-01-27

**Authors:** Glen M. Hocky, Charles V. Sindelar, Wenxiang Cao, Gregory A. Voth, Enrique M. De La Cruz

**Affiliations:** 1 Department of Chemistry, New York University, New York, New York, USA; 2 Department of Molecular Biophysics and Biochemistry, Yale University, New Haven, Connecticut, USA; 3 Department of Chemistry, Chicago Center for Theoretical Chemistry, Institute for Biophysical Dynamics, and James Franck Institute, University of Chicago, Chicago, Illinois, USA

**Keywords:** actin, cofilin, severing, molecular dynamics, computer modeling, cryo-EM, electron-cryomicroscopy, MD, molecular dynamics, RMSD, root-mean-square-deviation, SD, subdomain

## Abstract

Members of the ADF/cofilin family of regulatory proteins bind actin filaments cooperatively, locally change actin subunit conformation and orientation, and sever filaments at “boundaries” between bare and cofilin-occupied segments. A cluster of bound cofilin introduces two distinct classes of boundaries due to the intrinsic polarity of actin filaments, one at the “pointed” end side and the other at the “barbed” end-side of the cluster; severing occurs more readily at the pointed end side of the cluster (“fast-severing” boundary) than the barbed end side (“slow-severing” boundary). A recent electron-cryomicroscopy (cryo-EM) model of the slow-severing boundary revealed structural “defects” at the interface that potentially contribute to severing. However, the structure of the fast-severing boundary remains uncertain. Here, we use extensive molecular dynamics simulations to produce atomic resolution models of both severing boundaries. Our equilibrated simulation model of the slow-severing boundary is consistent with the cryo-EM structural model. Simulations indicate that actin subunits at both boundaries adopt structures intermediate between those of bare and cofilin-bound actin subunits. These “intermediate” states have compromised intersubunit contacts, but those at the slow-severing boundary are stabilized by cofilin bridging interactions, accounting for its lower fragmentation probability. Simulations where cofilin proteins are removed from cofilactin filaments favor a mechanism in which a cluster of two contiguously bound cofilins is needed to fully stabilize the cofilactin conformation, promote cooperative binding interactions, and accelerate filament severing. Together, these studies provide a molecular-scale foundation for developing coarse-grained and theoretical descriptions of cofilin-mediated actin filament severing.

The actin cytoskeleton is a dynamic biopolymer network that powers cell motility and division (1). The primary component of this network is the actin filament—a linear, helical, and polar polymer formed from the head-to-tail assembly of actin monomers. Actin filament assembly dynamics are controlled by a wide variety of regulatory proteins, among which are filament severing proteins that accelerate network turnover by increasing the concentration of polymer ends where subunits can add and dissociate (1).

Filament severing proteins in the cofilin/ADF family (herein referred to as cofilin) bind actin filaments between longitudinally adjacent actin subunits (2). A fully decorated ‘cofilactin” filament (having a stoichiometry of one cofilin per actin subunit (3)) is more compliant in bending (4, 5, 6, 7) and intersubunit twisting (7, 8) and also has a shorter helical repeat (9, 10) compared with a bare actin filament. Cofilin binding displaces a stabilizing intersubunit contact formed by the actin “D-loop” of one subunit and the “target binding cleft” of its longitudinally adjacent, pointed end neighbor (Fig. 1*A*) (11, 12, 13, 14). Cofilin maintains direct contact with both of these two subunits, forming a “bridge” that compensates for the loss of these intersubunit contacts (11, 12, 13, 14).

**Figure 1:**
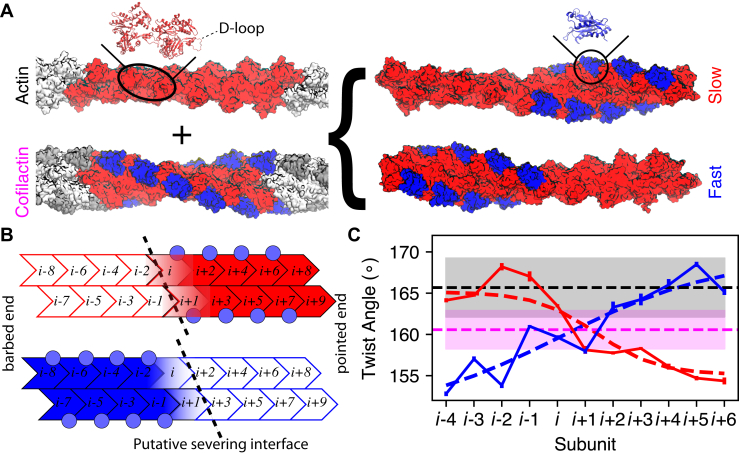
**Computationally generated cofilactin/actin boundary models are stable and exhibit abrupt changes in topology.***A*, MD-equilibrated periodic filament models on the left are combined to form two cofilactin–actin boundaries on the right (actin in *red*, cofilin in *blue*). Ribbon diagrams show the structure of (*left*) two longitudinal subunits interacting *via* a D-loop, and (*right*) one cofilin protein bound at the “bridging” site between the two subunits. The color scheme used in labeling the models—Actin (*black*), Cofilactin (*magenta*), Slow (*red*), and Fast (*blue*)—is continued in future figures when possible. *B*, Schematic of the two boundary models. Eighteen actin subunits are arranged in two “proto-filaments” with cofilin (*circles*) bridging two longitudinal adjacent actin subunits. Each of the “interfacial” subunits (*i* and *i*+1) contacts only one cofilin protein. A *dashed line* indicates the most likely location for severing (“putative severing interface”). *C*, Approximate relative twist angle between adjacent actin subunits computed from the last 50 ns of MD simulation. Horizontal *dashed lines* and *shaded areas* show the mean and standard deviation computed from the Actin (*black*) and Cofilactin (*magenta*) starting structures in (*A*). Filament twist transitions between a high and low twist value abruptly across the interfacial subunits (low to high for Fast (*blue*), high to low for Slow (*red*), with dashed lines showing fits described in Table 1).

Filaments partially decorated with cofilin sever at boundaries between bare and cofilin-decorated segments (15, 16, 17, 18, 19, 20), thereby explaining why fully decorated filaments are more stable than partially decorated ones (3, 15, 21, 22, 23, 24). Strained filaments can localize elastic energy at mechanical gradients such as those occurring at boundaries, which accelerates severing (25, 26, 27). However, filaments in solution that are partially decorated with cofilin also spontaneously fragment due to thermal fluctuations, indicating that the boundary interface is less stable than actin–actin or cofilactin–cofilactin interfaces (6). The two boundaries of a cofilin cluster are not structurally identical, and it has been shown that the barbed end side severs at a lower rate than the pointed end side (18, 28). An intermediate resolution structure (subnanometer) of the slow-severing cofilactin/actin boundary was recently solved by electron cryo-microscopy (cryo-EM), but even this level of detail has not been achieved for the fast-severing boundary (29).

Molecular dynamics (MD) simulations have been successful in capturing the molecular details and dynamics of actin filaments, including cofilin-linked changes to structure and filament rigidity (5, 7, 12, 13, 30). Here, we employ MD simulations to predict structures of these two boundaries starting from the bare actin and cofilactin filament structures. Our simulations are consistent with known data for the slow-severing boundary, predict an intermediate state of actin subunits within both interfaces, and provide a structural basis for the asymmetric severing of filaments by cofilin. Further simulations of synthetic boundaries generated by removal of cofilin from cofilactin structures allow us to assess stability of cofilin clusters of varying size.

## Results and discussion

### *In silico* cofilactin filament models

Initial periodic structures for bare and cofilin-decorated filaments (herein referred to as cofilactin) were generated from EM structures of ADP-actin (PDB 2ZWH) and cofilactin (PDB 3J0S) (11, 31) (see Simulation Methods), followed by all-atom MD. The resulting, equilibrated structures (Fig. 1*A*) were joined end to end in a head-to-tail manner (*e.g.*, the barbed end of one filament was placed adjacent to the pointed end of the other) through alignment of two subunits from each structure (details in Simulation Methods), yielding starting models for the slow- and fast-severing boundaries (Fig. 1*A*). Extensive additional MD simulations were then performed and analyzed as described below. The resulting systems contain cofilactin segments with bare actin at either the pointed end (herein referred to as the “fast-severing” boundary) or barbed end (herein referred to as the “slow-severing” boundary) of the bound cofilin cluster (Fig. 1, *A* and *B*).

### Modeled filament boundaries are stable on the 100 ns timescale

Both fast-severing and slow-severing boundaries of modeled filaments appear to stabilize within the first 25–30 ns of the MD simulations, as indicated by the root-mean-square-deviation (RMSD) of the actin subunits at the boundary (subunits *i-*2: *i+*1 in Fig. 1*B*); filaments remain stable and do not undergo significant further structural rearrangements for >150 ns, after which slower-timescale rearrangements occur on the <2 Å length scale (Fig. S1). A fast-severing boundary model lacking intersubunit D-loop contacts was not stable, resulting in partial filament rupture (Fig. S1; described in Simulation Methods).

**Figure 2:**
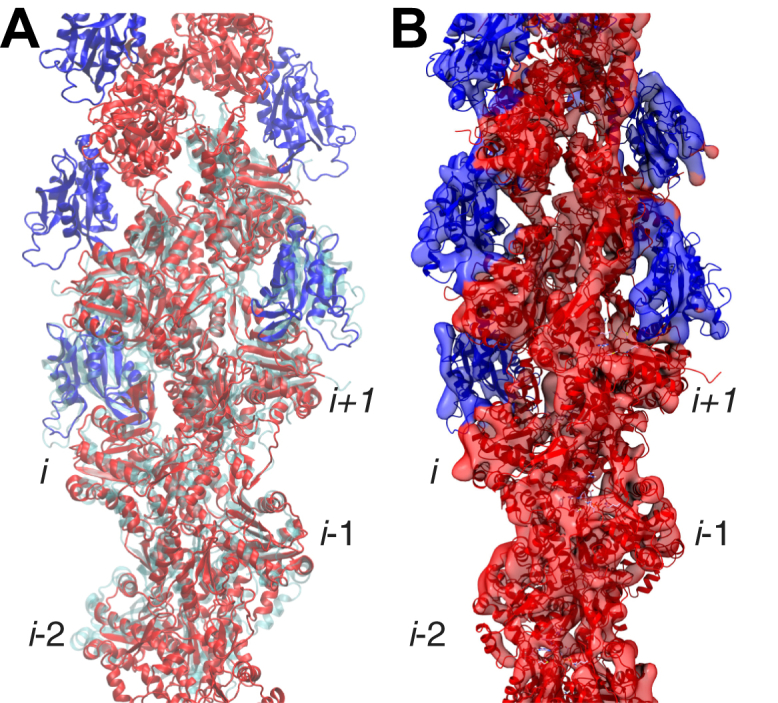
**Molecular dynamics model of slow-severing boundary is consistent with cryo-EM data.***A*, Alignment of actin subunits *i*-2: *i*+1 from the final Slow boundary model structure, *red* and *blue*, to that from PDB ID 6UC4, semitransparent *blue* (29), shows good agreement for the structure of the interface, as quantified in Fig. S2. *B*, This boundary model structure is also consistent with the original ∼9 Å resolution cryo-EM density map (EMDataBank EMD-20726). Here, the same structure in A is shown with the EM map overlaid (semitransparent isosurface), after performing an all-atom alignment of the full MD structure to the density using UCSF Chimera (32).

The atomic structure of our slow-severing boundary model is consistent with a previously reported ∼9 Å resolution cryo-EM map (Fig. 2) (29). A boundary model had been generated from that map by rigid body docking of prior actin and cofilactin subunit structures into the electron density. Our MD model is in reasonable overall agreement with this structure (Fig. 2), as quantified by either the RMSD of “boundary subunits” (*i*-2: *i*+1) or the “interfacial subunits” (*i* and *i+*1) (Fig. S2). The “flatness” of these subunits also agrees well with the cryo-EM model (see next section; Fig. S3). The data from Ref. (29) is not of high enough resolution to evaluate the precise details of our simulation model beyond these comparisons, but the level of agreement between our simulations and the assumed molecular structure lends confidence to our approach and supports the utility of MD to predict features of the fast-severing interface, for which no high resolution structure is available.

### Subunit and filament conformations change abruptly at actin–cofilactin boundaries

Cofilin binding changes the helical twist of actin filaments by ∼5 degrees (on average) per subunit (167° → 162° as measured by cryo-EM (10) or 166° →161° by MD (7). In our simulations, the filament twist changes from the cofilactin value to the bare value (and vice versa) over the course of 1–3 subunits at both the fast- and slow-severing boundaries, with a slightly shorter crossover length at the slow-severing boundary than the fast-severing boundary (Fig. 1*C*, Table 1), consistent with recent cryo-EM analysis (34).

**Table 1:** Twist angles (Fig. 1*C*) and internal flattening angles (Fig. 3) are fit to a transition model as in Ref (33, 34).

System-observable	*A*_1_ (barbed side angle, °)	*A*_2_ (pointed side angle, °)	*n*_0_ (central position)	*N* (crossover length)
**Slow-twist**	165.1	155.1	1.4	1.1
**Fast-twist**	150.0	169.4	0.1	2.9
**Slow-*ϕ***	−9.0	−28.9	−0.3	1.1
**Fast-*ϕ***	−29.4	−4.2	2.2	2.8

The fit function used for the angle as a function of position *n* is $\theta \left(n\right)=A_{2}\;-\;\frac{A_{2}-A_{1}}{1\;+\;\text{exp}(n-n_{0})/N}$ where n refers to subunit position *i+n* (n from –8 to 9 in Fig. 1*B*), N is the exponential decay length (crossover length), n_0_ is the center of exponential decay or crossover, and A_1_ and A_2_ are limits of the function for either twist or flattening angles across a boundary. The result of the fit for the dihedral angles is shown in Fig. S3.

**Figure 3:**
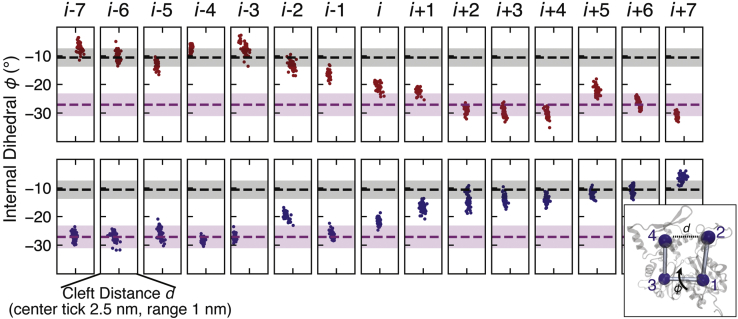
**Internal actin subunit configuration changes abruptly at the actin/cofilactin boundary.** (*Inset*) Ribbon diagram of an actin monomer with beads corresponding to the center of mass of actin’s four primary subdomains (SD), as defined in Ref. (35). Cleft distance *d* and the flatness angle *ϕ* (an effective torsion angle with respect to the centers of the four subdomains) can characterize different actin configurations. (Main figure) Scatter plot of *ϕ* versus *d* for subunits in the slow-severing (*top*, *red*) and fast-severing (bottom, *blue*) filament models (final 50 ns of simulation). *ϕ* changes abruptly at the boundary (see also Fig. S3) between actin-like range (*black dashed line* = mean, one SD is shaded) and a cofilactin-like range (*magenta*). NB: The flattening angle *ϕ* is not to be confused with the third Euler angle, commonly denoted with the same Greek letter ‘*φ*’, used in cryo-EM image analysis to define the “twist” (axial orientation of a helical subunit with respect to the filament axis).

In addition to altering the helical twist, cofilin binding also tilts the outer domain of filament subunits such that bare actin subunits are “flatter” than those in cofilactin (11). Therefore, actin subunit “flatness” serves an additional proxy for assessing cofilin-induced structural changes, quantified here by the dihedral angle *ϕ* of the four major actin subdomains (SDs; Fig. 3, inset). Actin subunits within bare and cofilin-decorated regions maintain their initial subunit flatness, as indicated by *ϕ* values that remain near the canonical values (horizontal dashed lines in Fig. 3). In contrast, “interfacial subunits” *i*, *i+*1 adopt a *ϕ* value intermediate between that of bare actin and cofilactin (Fig. 3). The abrupt change in twist and subunit conformation at cofilactin boundaries observed in our MD models provide further evidence that actin structural changes linked to cofilin binding are local, propagating only to nearest neighbors directly in contact with cofilin (3, 15, 16, 19, 29, 34).

### Structures of the slow- and fast-severing boundaries

Interfacial actin subunits (subunits *i* and *i*+1) adopt unique structures intermediate between that of subunits in bare actin and cofilactin. As a consequence of this intermediate structure induced by cofilin binding at either the pointed end (slow-severing) or barbed end (fast-severing) of the actin subunits, we expect a change in the nature of the interactions between subunits at the boundary.

**Figure 4:**
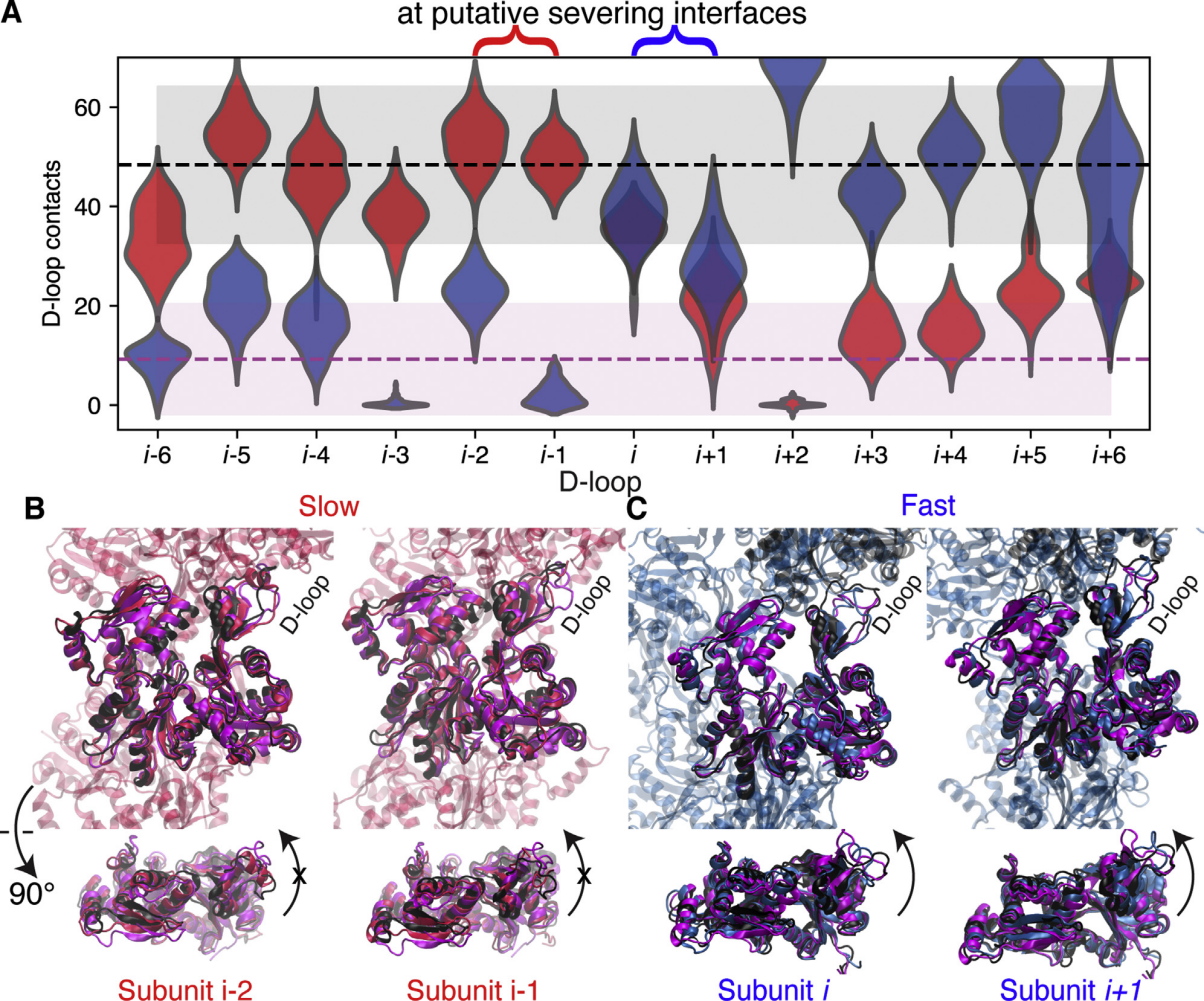
**Subunits at fast-severing boundary lack D-loop contacts due to intermediate internal twist.***A*, Violin plot (*blue* and *red* for the fast- and slow-severing models, respectively) showing a histogram of D-loop contacts (number of C_⍺_ distances < 1 nm), symmetrically reflected, at each subunit position. The number of D-loop contacts of an interfacial subunit *i* or *i*+1 with the longitudinally adjacent subunit at its pointed end is intermediate between an actin-like range (*light gray*) and cofilin-like range (*light magenta*) for both the fast- and slow-severing models. Curly braces above indicate the location of the putative severing interface (Fig. 1). *B* and *C*, Structures are actin subunits from the slow-severing (*red*; *B*) or fast-severing (*blue*; *C*) models’ putative severing interfaces. Overlaid are subunits from the same simulation in the bare region (*black*) and cofilactin region (*magenta*). The D-loops are the labeled unstructured region at the top right in all four upper panels. In (*B*), the *red* D-loops are more extended and in contact with the next subunit above in contrast to (*C*), where the blue D-loops are displaced in a more cofilactin-like manner, consistent with the measurements shown in (*A*). In the *lower panels* of *B* and *C*, the central subunits are rotated so that the subunit flatness can be observed. In (*B*), the red subunit conformations are fairly flat, more similar to the bare actin (*black*) configuration, whereas in (*C*) the blue subunit conformations are more twisted and similar to the cofilactin configuration (*magenta*).

In Figure 4*A*, we show the distribution of the number of D-loop contacts in an actin subunit with its longitudinal neighbor at the pointed (right) end. As a consequence of the intermediate configurations adopted at the interfacial subunits, intersubunit D-loop contacts and other longitudinal contacts at the pointed end side (Fig. S4*A*) are compromised for both boundary models (contacts between subunits *i*: *i*+2, *i*+1: *i*+3). This disruption occurs without formation of additional lateral contacts (Fig. S4*B*). However, in the case of the slow-severing model, the reduction in longitudinal filament contacts is compensated by the stabilizing cofilactin “bridge” interactions (Fig. S4*C*), with a cofilin nestled between SD2 of the actin subunit at its barbed end side and SD1 of the actin subunit at its pointed end side (see ribbon diagram in Fig. 1*A*) (24). Hence, the pointed end of subunits *i,i+*1 is less likely to be a locus of severing at the slow-severing boundary than at the fast-severing one.

Indeed, our prediction is that filament fragmentation is most likely to occur where bare actin-like subunits contact the interfacial subunits (“putative severing interface” in Figs. 1*B* and 4A). This location is asymmetric with respect to the fast- and slow-severing models, since it occurs at the barbed end of the interfacial actin subunits in the slow-severing case (interface *i*-2:*i*, *i*-1:*i*+1) and at the pointed end in the fast-severing case (interface *i*:*i*+2, *i*+1:*i*+3). For the fast-severing model, this putative severing interface is coincident with the location of a pronounced reduction in D-loop contacts (Fig. 4*A*), consistent with our hypothesis that this interface is structurally weak and more prone to failure than those between other subunits. In contrast, we do not observe a substantial reduction in D-loop contacts at our predicted slow-severing location (Fig. 4*A*); this greater total number of contacts at the D-loops of actin subunits *i-*2, *i-*1 is consistent with the slower severing observed experimentally (18, 28). However, the interface between bare actin and cofilactin is still the most plausible location for severing due to (1) the abrupt change in structure of the subunits, (2) a reduction in lateral contacts (subunit *i*-2, Fig. S4*B*), and (3) the intermediate flattening observed at subunits *i-*2*, i-*1 (Fig. 3) (29).

Finally, we note that while the reduction in D-loop contacts at the fast-severing interface *could* be a consequence of model construction, these D-loop interactions were required for a stable model (see Simulation Methods). As noted in describing model construction, the filament partially ruptured unless a biasing potential was used to drive interfacial D-loops within interacting distance of their longitudinal neighbors. Despite this biasing potential, D-loops of interfacial subunits at the fast-severing boundary failed to fully restore actin-like interactions with their longitudinal neighbors—for example, not wrapping around the Y169 as seen in experimentally determined actin filament structures (36, 37). Our data suggest that bridging cofilin interactions at the barbed end of interfacial subunits at the fast-severing boundary allosterically restrict the ability to adopt actin-like conformations and corresponding D-loop contacts at the putative fast-severing interface (pointed end of the interfacial subunits).

### Filament conformational changes following computational ablation of cofilin

**Figure 5:**
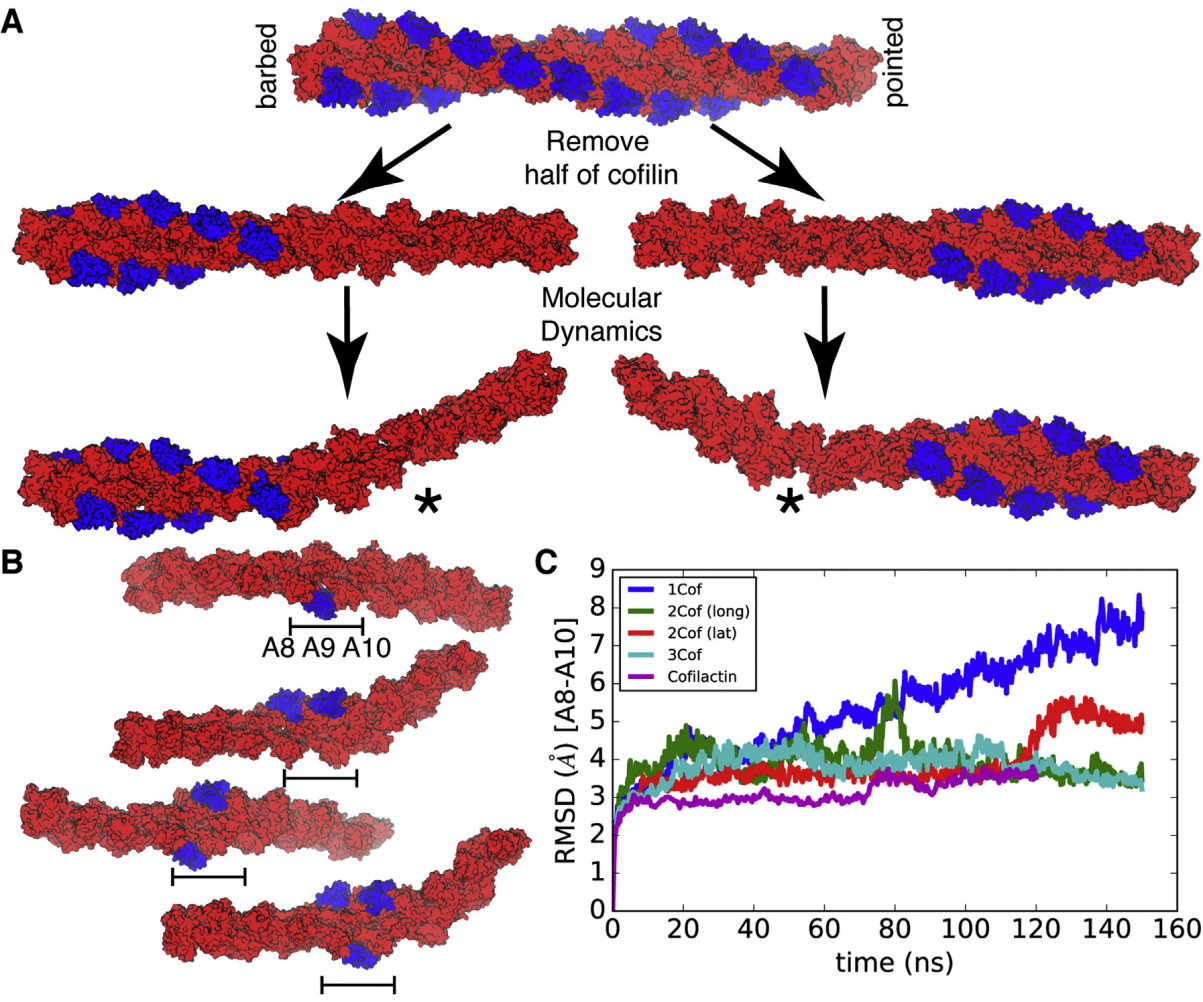
**Computational ablation of cofilin shows short ranged effect of cofilactin domain and minimal cluster size of two cofilins.***A*, Computational ablation procedure results in stable cofilactin domains and unstable bare actin domains, including “severing” of protofilament, indicated by “∗” (*bottom* are final structures from movies Movie M.1, Movie M.2). In images for (*A*) and (*B*), shading indicates depth. *B*, Final structures after ablating all but n = 1–3 subunits. The second structure has two cofilins arranged longitudinally, while the third has two arranged laterally. *C*, Graph shows all-atom RMSD compared with initial cofilactin structure of three actin subunits indicated by a bar in *B*. The subunits in n = 2,3 systems are almost as stable as original cofilactin structure while for n = 1, the configuration changes rapidly.

Simulations of “cofilin-ablated” filaments further support the notion that a reduction in intersubunit D-loop contacts contributes to changes in filament compliance and severing. We developed an alternative strategy to examine boundaries whereby we simulated a cofilactin filament after computationally “ablating” a fraction of initially bound cofilins, resolvating and equilibrating (Fig. 5*A*; see also Simulation Methods). The segments with cofilin removed, as initialized, have a drastically reduced number of D-loop contacts relative to a bare actin filament and undergo thermally driven, nonequilibrium bending fluctuations that far exceed those of fully occupied cofilactin filaments (Movie M.1–Movie M.3).

Further, the reduction of intersubunit contacts in the cofilin-ablated region led to hinge-like bending adjacent to the interfacial subunits where the contacts are only partially compromised (Movie M.1 and Movie M.2). The flexible zone in both slow- and fast-severing cases extends beyond the “hinge” region to include all actin subunits in the cofilin-ablated segment. In both simulations, a partial rupture occurred in the high-flexibility (cofilin-ablated) zone, severing the protofilament at one place (marked by “∗” in Fig. 5*A*). In contrast, cofilin-bound subunits (including the interfacial subunits) retain cofilactin conformations, as measured by their lower *ϕ* values, albeit with significantly larger variance (Fig. S5). Although the way in which these simulation models were created is not physiological, the observed hinge-like motion has been observed at boundaries and linked to a higher severing probability (6).

### Small cofilin clusters (n ≥ 2) retain the cofilactin conformation

Small, bound cofilin clusters (n = 2 or 3; Fig. 5) formed by computational ablation were found to be stable, as shown by computing the RMSD of actin subunits within the cluster to the starting model (over the course of 160 ns of MD simulations following ablation; Fig. 5*C*). In contrast, ablation of all but a single cofilin does not retain the cofilactin structure (Fig. 5*C*). In the corresponding simulation (n = 1), the average RMSD to the starting model increases soon after the start and continues to increase for the duration of the simulation although the cofilin remains stably bound (Fig. 5*C*). This behavior suggests that two contiguously bound cofilin molecules, either longitudinal or lateral, are sufficient to retain the cofilactin conformation and are consistent with a cooperative binding nucleus size of two contiguously bound cofilins (29).

## Conclusions

A model of the slow-severing actin–cofilactin boundary (barbed end of a cofilin domain) constructed and observed by MD captured critical structural features of the interface that was recently determined at intermediate resolution by cryo-EM. This consistency lends credence to a computational model of the fast-severing boundary (pointed end of a cofilactin domain), which has not been visualized by cryo-EM. The proposed severing location for the modeled fast-severing boundary (pointed end of the interfacial subunits) exhibits compromised D-loop and other longitudinal contacts without compensatory stabilizing cofilin interactions, commensurate with enhanced severing at that boundary. While the same location in the slow-severing boundary has a reduction in longitudinal contacts at interfacial subunits, similar to what is seen at the fast-severing boundary, this is compensated by contacts with a bound cofilin at each subunit and hence is not the most likely locus for severing. Instead, we propose that the severing interface for the slow-severing boundary is at the barbed end of the interfacial subunits due to the abrupt change in structure. However, the higher number of contacts at the position we deem most likely for severing is consistent with a slower-severing rate compared with the severing location (interface) for the fast-severing boundary (pointed end of interfacial subunits).

Our simulation models agree with prior data showing that conformational changes are highly localized and propagate asymmetrically, extending 2–3 subunits at the fast-severing boundary versus 1–2 at the slow-severing boundary (Table 1) (34). These features offer a further structural explanation for the asymmetric severing (18, 28) and growth (18, 38, 39) at the two cofilactin cluster boundaries (*i.e.*, at pointed end or barbed end side).

In this work, we also introduce computational ablation of cofilin proteins as a way to evaluate the behavior and stability of cofilactin domains. While this is a computational experiment that cannot be performed in “wet-lab” experiments, it allowed us to gain new insights into cofilin cluster stability. These data support the idea of a minimal domain size of two cofilin and the very short ranged propagation of the cofilactin twist away from the boundary.

In summary, a wide range of cofilactin severing models implicate alterations in filament mechanical properties and dynamics (4, 13, 26, 27, 40, 41) in addition to structure (5, 7, 10, 11, 14, 29, 34). These aspects are undoubtedly linked, but the molecular origins of the coupling had not been previously established. The reduction in longitudinal filament contacts at the severing interface revealed here, particularly those mediated by the actin D-loop, provides a link between filament structure, mechanics, dynamics, and severing.

### Simulation methods

Actin filament structures with bound ADP and cofilactin bound structures were built and equilibrated at 310 K as in prior work (13, 30, 35, 42). The structure of ADP-bound actin derived from the electron microscopy structure in PDB ID 2ZWH and for cofilactin from 3J0S (11, 31). In each actin subunit, the nucleotide was replaced by a magnesium and water-coordinated ADP molecule from a previously equilibrated monomer simulation. MD simulation of these structures was then performed using GROMACS (43). A cofilin-decorated filament with 11 subunits was equilibrated using ∼5 ns of MD (and remained close to the initial structure from 3J0S). Structures for bare filaments were taken after 40 ns of MD simulation performed for a 26-subunit actin filament. These structures are shown in Figure 1*A* (*left*). Periodic boundary conditions are employed in all simulations, and the size of the simulation box in the case of the “Actin” and “Cofilactin” models is commensurate with the periodic repeat length of the filament, such that those models are somewhat akin to an infinite length filament (Fig. 1). In contrast, all other systems have nonperiodic filament geometries, and hence extra water padding is used between the barbed and pointed ends in those cases.

All systems are simulated using CHARMM22+CMAP and solvated in TIP3P water, including 180 mM KCl plus neutralizing potassium ions as in our past MD studies (13, 30, 35, 42). The water box for nonperiodic filaments is padded by 2.4 nm of water for the structures in Figure 1 and at least 3 nm of water in the lateral directions and 4 nm of water in the longitudinal direction for the structures in Figure 5. Interface simulation models in Figure 1 contain approximately 1 million atoms, while those in Figure 5 contain approximately 1.3 million atoms. Full simulation parameters as well as initial, final, and intermediate structures of all models built in the next section are available in a GitHub repository for this project (https://github.com/hocky-research-group/CofilinSevering).

### Construction of fast- and slow-severing interfaces

The slow-severing boundary (Fig. 1, “Slow”) was constructed by aligning one actin from the 26-subunit “Actin” filament (equivalent to actin *i*, Fig. 1) with one actin from the “Cofilactin” structure (also actin *i*) by minimizing the RMSD of C_α_ atoms. The final interface model consists of eight actin subunits (*i*-8 to *i*-1) from the Actin structure and ten actin subunits from the Cofilactin structure (*i* to *i*+9), which includes the one that was aligned. Additionally, eight cofilins from that structure within the cofilactin domain were retained. Hence the interfacial actin starts in a cofilactin-like state. The combined structures were then solvated, ionized, and equilibrated by the procedure in Ref. (30), followed by an additional 310 ns of MD simulation.

The fast-severing boundary (Fig. 1, “Fast”) was constructed in a similar manner, but more care was needed to produce a stable model, due to the initial dearth of longitudinal contacts after structural alignments. The positions of 12 actin subunits in a cofilactin structure and ten from an actin structure were used. The inner subdomains (SD 3 and 4) of four actins at the pointed end of the cofilactin model and four actins from barbed end of the bare actin model were aligned (equivalent to subunits *i* to *i*+3). The first ten actin subunits (*i*-8 to *i*+1) and associated eight cofilins from the cofilactin model were retained, as well as the final eight actin subunits from the actin structure (*i*+2 to *i*+9). Again, the structure is then prepared for simulation by the procedure in Ref. (30). This boundary model with only longitudinal contacts in the inner domains (and little to no D-loop contacts produced from this alignment) was not stable and quickly formed a kinked structure reminiscent of severing (Fig. S1*B*). To restore some longitudinal contacts without prescribing any particular structure, we then applied a moving harmonic bias with spring constant 50 kcal/(mol Å^2^) between the center of mass of D-loop C_α_ atoms (residues 44–52) and target binding cleft residues (143–148, 349–351) for the two interfacial atoms, with the MOVINGRESTRAINT function of the PLUMED library (44). The center of this harmonic restraint moved from the initial distances (2.5 and 2.7 nm) toward a rough estimate of the bare actin distance (1.7 nm) over 10 ns. This restraint was then maintained for an additional 30 ns. After that, we performed the same 310 ns of MD simulation as for the previous case.

An alternative procedure for generating the Fast boundary was also performed equivalent to the generation of the Slow interface, aligning actin subunit *i* and retaining eight actin subunits from the cofilactin structure (*i*-8 to *i*-1) and ten from bare actin (*i* to *i+*9), such that the interfacial actin started in an actin-like structure. This resulted in a stable model, however, one in which the interfacial actin never evolved away from an initially actin-like structure in response to the presence of associated cofilin at their barbed end (Fig. S1). Because this contradicted evidence from experimental data (29), we did not analyze this model further.

### Construction of interfaces by computational ablation

The 11-subunit Cofilactin structure was replicated once in the longitudinal direction, producing a 22-subunit cofilactin structure containing 20 cofilins. Initial structures of systems shown in Figure 5 were created by simply not including selected cofilin proteins in the generated structures. Each structure was re-equilibrated by the procedure in Ref. (30). For the two interfaces in Figure 5*A*, harmonic restraints of 1000 kJ/(mol nm^2^) were applied to the backbone atoms of the two actins and two cofilins at the cofilin-decorated end of the filament, to represent an infinite extension of the system in that direction. These restraints also prevented the large initial rearrangements from rotating the filament outside of the oblong box, causing unphysical self-interactions. For the simulations in Figure 5*C*, only 17 actins were used to reduce the simulation cost.

## Data availability

Structural data on cofilin interface models is available from the author’s GitHub repository for this paper: https://github.com/hocky-research-group/CofilinSevering. All other data required for the conclusions made here are contained within the article or Supporting Information. Any other data is available at request from the authors.

## Conflict of interest

The authors declare that they have no conflicts of interest with the contents of this article.
