# Supplementary material for: Structural basis of fast- and slow-severing actin–cofilactin boundaries
Source: J Biol Chem. 2021 Jan 27;296:100337. doi: 10.1016/j.jbc.2021.100337 (PMC7961102; doi:10.1016/j.jbc.2021.100337)
Supplement: Figures S1–S5 [file mmc1.pdf]

Supporting information for:

Structural basis of fast- and slow-severing actin–cofilactin boundaries

Glen M. Hocky,[1,*] Charles V. Sindelar,[2] Wenxiang Cao,[2] Gregory A. Voth,[3] Enrique M. De La Cruz[2,*]

1. Department of Chemistry, New York University, New York, NY
2. Department of Molecular Biophysics and Biochemistry, Yale University, New Haven, CT, USA
3. Department of Chemistry, Chicago Center for Theoretical Chemistry, Institute for Biophysical Dynamics, and James Franck Institute, University of Chicago, Chicago, IL

*Corresponding author's emails: hockyg@nyu.edu, enrique.delacruz@yale.edu

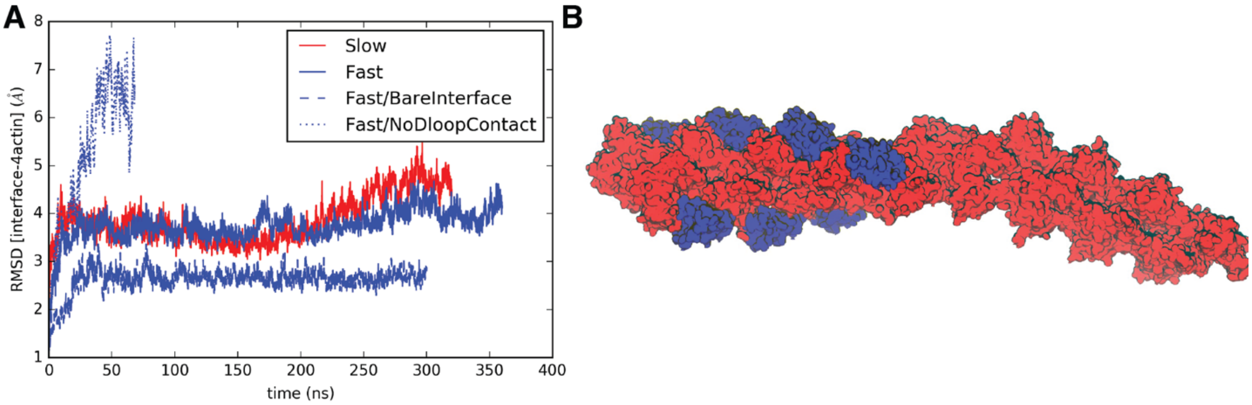

Fig. S1. (A) RMSD of *i*-2,*i*-1,*i*,*i*+1 subunits for different boundary models generated in this study, with the Slow and Fast models from the main text in solid lines. The dotted line shows an unstable structure similar to the Fast boundary model but formed without adhering D-loops described in the main text. The dashed line (bottom curve) shows a much more stable structure similar to the Fast boundary model where *i*,*i*+1 actin start in an actin-like rather than a cofilactin like configuration. (B) Final structure of the unstable fast-severing model in (A), simulating without first adhering D-loops.

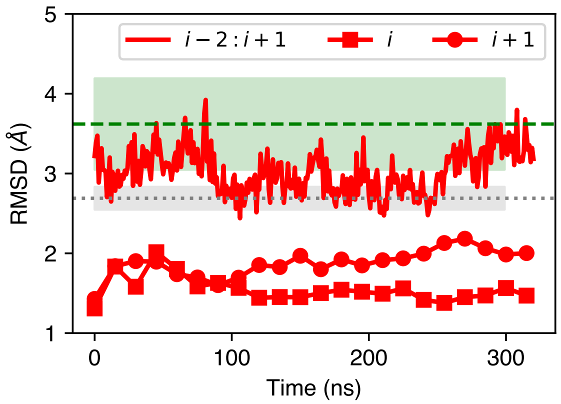

Fig. S2. Comparison of the Slow boundary model to the model created by rigid body fits to an electron density map in Ref. (29) (see Fig. 2). The thick red line shows C_α RMSD of the four subunits at the boundary (*i*-2,*i*-1,*i*,*i*+1) during the course of the MD simulation for the residues 5-38 and 64 to 370 (not all residues are included in the deposited structure). The green dashed line and shaded box show the average and one standard deviation of the RMSD of these four subunits to themselves within the MD simulation using the same residues. The red square and circle lines show the RMSD of a single interfacial subunit between the MD and EM models, subunits *i* and *i*+1 respectively. The grey dashed line and shaded area show the RMSD and standard deviation of a single actin subunit to a cofilactin subunit in the MD simulation (comparing subunit *i*-5 to subunit *i*+6) as a reference.

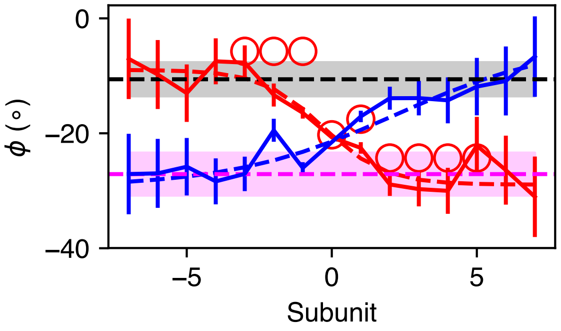

Fig. S3. Dihedral twist values from Fig. 3 fit to the transition model described in Tab. 1. As in Fig. 3, horizontal dashed lines and shaded areas show the mean and standard deviation computed from the Actin (black) and Cofilactin (magenta) starting structures. Filament twist transitions between a high and low twist value abruptly across the interfacial subunits (low to high for Fast (blue), high to low for Slow (red), with dashed lines showing the result of the fit). Open red circles show the angle values calculated from the model in Ref. (29), a rigid body fit to the electron density.

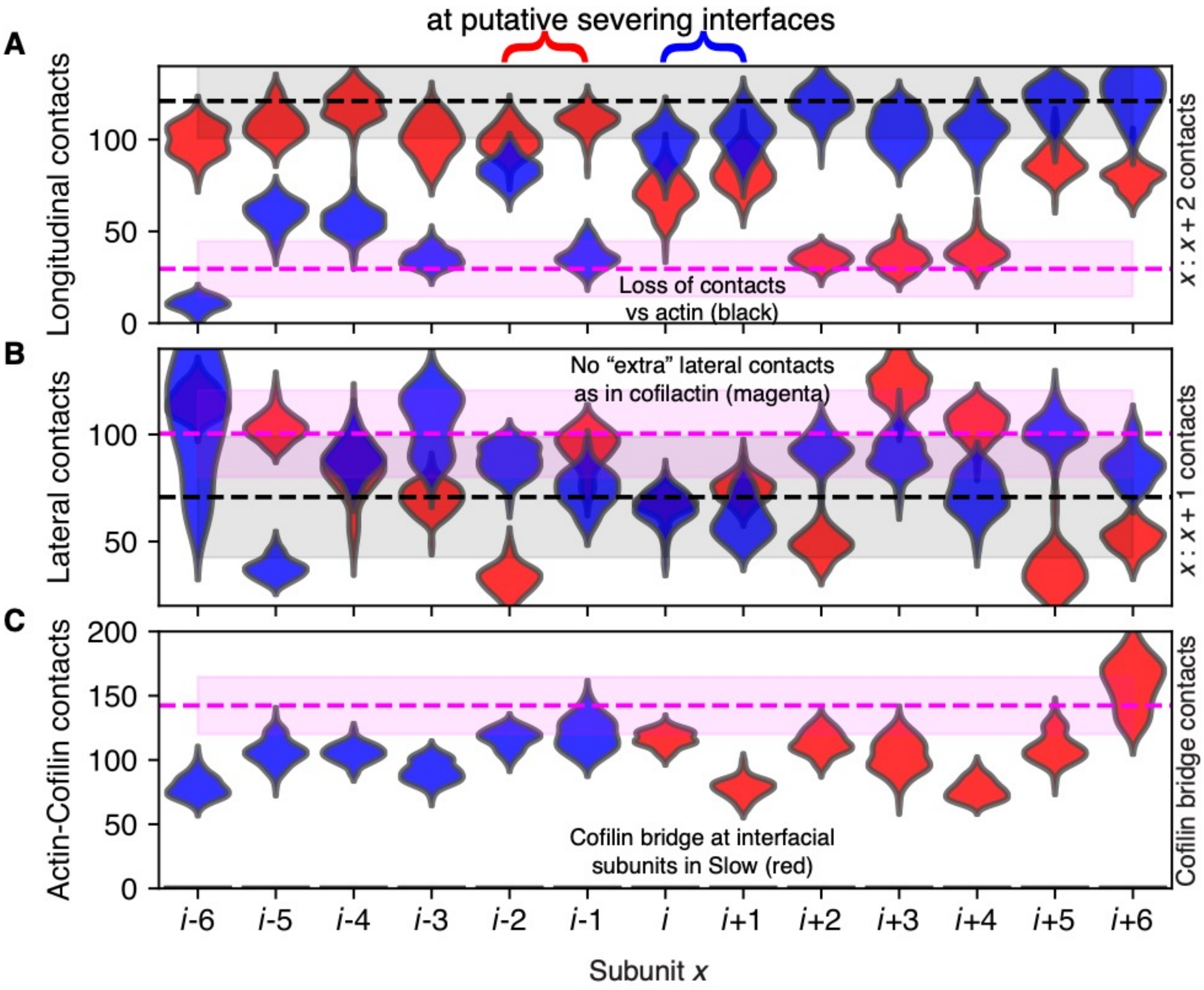

Fig. S4. Violin plots of contacts as in Fig. 4. (A) Violin plot of all longitudinal contacts (those between subunit *x* : *x*+2). As in other figures, the Slow interface is in red, and Fast interface is in blue. For Slow, the cofilin bound subunits are on the right end of the figure, and hence we expect fewer longitudinal contacts at that end, closer to the cofilactin average (magenta dashed line). For Fast, we expect reduced longitudinal contacts on the left end of the figure. (B) Lateral contacts (those between subunit *x* : *x* + 1). As discussed previously (7), cofilactin has slightly more lateral contacts than bare actin (magenta dashed line vs black dashed line). We might wonder if lateral contacts would be at or exceed the cofilactin value at the interfacial subunits (*i*, *i*+1) to compensate for the reduced longitudinal contacts in the upper panel; however, they instead show more bare actin-like values in our simulation. We note that one of the subunits in the putative severing interface of the Slow system (at position *i*-2 for *i*-2 : *i*-1 contacts) has significantly reduced lateral contacts. (C) Cofilin bridge contacts between an actin subunit and the cofilin to its pointed end. These contacts are only present within the cofilactin domain, and in our simulations are slightly reduced as compared to the fully decorated Cofilactin system (magenta dashed line), which could indicate some moderate longer-ranged structural disruption due to the presence of the boundary or finize size effects due to using short filaments. The Slow system has bridging contacts at the interfacial position (*i* and *i*+1) where longitudinal contacts are reduced.

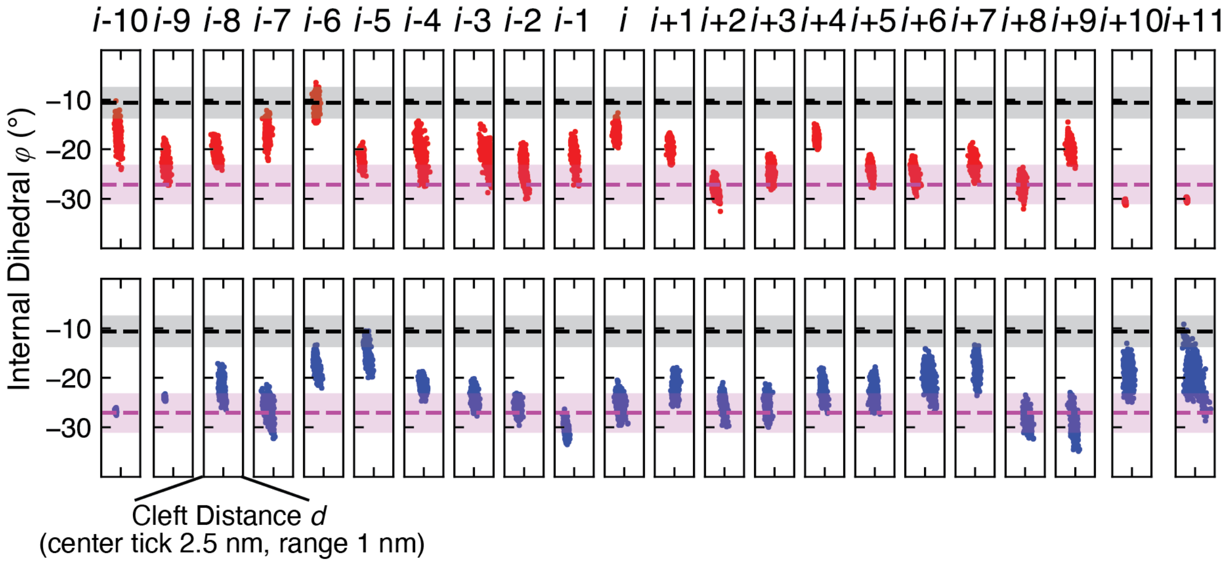

Fig. S5. Flattening dihedral angle and cleft distance as in Fig. 3, but for the last 100 ns of slow- (upper; red) and fast- (bottom; blue) severing boundaries generated by computational ablation (Movie M.1, Movie M.2). Although there is no bound cofilin on the left and right ends of the plots, respectively, the angles in these bare regions do not relax all the way to the bare actin value (black shaded area). As discussed in Simulation Methods, there are restraints placed on the final two cofilactin subunits, resulting in the small dots seen in the far right of the top graph and far left of the bottom graph.

Supplemental Movies

Movie M.1. Movie of the first 400 ns of molecular dynamics following the generation of a slow-severing boundary by computational ablation. Hinge-like motion is observed at the boundary between cofilin bound and bare actin. A severing-like event takes place, as indicted by a '*' in Fig. 5A.

Movie M.2. Movie of the first 390 ns of molecular dynamics following the generation of a fast-severing boundary by computational ablation. Hinge-like motion is observed at the boundary between cofilin bound and bare actin. A severing-like event takes place, as indicted by a '*' in Fig. 5A.

Movie M.3. This movie shows 120 ns of molecular dynamics for a large non-periodic cofilactin segment (22 actin, 20 cofilin with water added at both ends of the box as described in Simulation Methods). This illustrates the amount of flexibility expected for a cofilactin segment, which is much smaller than that observed following ablation.
